# An education intervention to improve decision making and health literacy among older Australians: a randomised controlled trial

**DOI:** 10.1186/s12877-019-1143-x

**Published:** 2019-05-07

**Authors:** Caroline A. Smith, Esther Chang, Gisselle Gallego, Afshan Khan, Mike Armour, Lynda G. Balneaves

**Affiliations:** 1 NICM Health Research Institute, Western Sydney University, Locked Bag 1797, Penrith, NSW 2571 Australia; 2 School of Nursing and Midwifery, Western Sydney University, Penrith, NSW Australia; 3 School of Medicine, The University of Notre Dame, Sydney, NSW Australia; 4 College of Nursing, Rady Faculty of Health Sciences, University of Manitoba, Manitoba, Canada

**Keywords:** Randomised controlled trial, Health literacy, Complementary medicine, Decision making

## Abstract

**Background:**

National policies seek to involve older Australian’s in decisions regarding their care; however, research has found varying levels of decision self-efficacy and health literacy skills. An increasing number of older Australians use complementary medicine (CM). We examined the effectiveness of a CM educational intervention delivered using a web or DVD plus booklet format to increase older adults’ decision self-efficacy and health literacy.

**Methods:**

A randomised controlled trial was conducted. We recruited individuals aged over 65 years living in retirement villages or participating in community groups, in Sydney Australia. Participants were randomly allocated to receive a CM education intervention delivered using a website or DVD plus booklet versus booklet only. The primary outcome was decision self-efficacy. A secondary outcome included the Preparation for Decision-Making scale and health literacy. Outcomes were collected at 3 weeks, and 2 months from baseline, and analysed using an adjusted ANOVA, or repeated measures ANOVA.

**Result:**

We randomised 153 participants. Follow up at 3 weeks and 2 months was completed by 131 participants. There was a 14% (*n* = 22) attrition rate. At the end of the intervention, we found no significant differences between groups for decision self-efficacy (mean difference (MD) 3.8, 95% confidence interval (CI) -2.0 to 9.6 *p* = 0.20), there were no differences between groups on nine health literacy domains, and the Preparation for Decision-Making scale. Over 80% of participants in both groups rated the content as excellent or good.

**Conclusion:**

Decision self-efficacy improved for participants, but did not differ between groups. Decision self-efficacy and health literacy outcomes were not influenced by the delivery of education using a website, DVD or booklet. Participants found the resources useful, and rated the content as good or excellent. CM Web or DVD and booklet resources have the potential for wider application.

**Trial registration:**

The trial was registered with the Australian New Zealand Clinical Trials Registry: ACTRN (ACTRN12616000135415). The trial was registered on 5 February 2016.

## Background

The aging population in Australia is rising. In 2016 15.8% of Australians were aged over 65 years [1]. Complementary medicine (CM) is widely used in Australia, and 58% of Australians aged over 65 years report use of a CM modality in the previous 12 months [2]. In this population, CM is reported to treat conditions associated with aging [3], including musculoskeletal conditions and pain [4], as well as depression and anxiety [5]. The use of CM in this population support previous study findings that older people actively engage in their health care to improve their health and wellbeing [6], and CM provides an avenue for individuals to participate in their health and self-care [7]. Older people using CM report significant mental and physical health benefits from CM [8, 9].

Self-efficacy, also referred as personal efficacy, is confidence in one’s ability to improve the quality of health decision making. However, it is unclear whether the use of decision-making resources or aids do improve the quality of health decision for the older person. Older adults making health decisions are often seen as complex and it is unclear whether the impact of the results of using decision making resources do help older people or give them more self-efficacy [10]) and in making better decisions. Decision resources or aids are often intended to assist older people to examine and weight the benefits and harms or problems in treatments [11].

A systematic review and meta-analysis conducted by van Weert et al. (2016) [10] presented encouraging results on the effectiveness of decision aids for older adults. Although the review showed decision aids improved older adults’ knowledge, increase their risk perception and decrease decisional conflict, only one decision resource or aid was developed for older adults. Furthermore, the authors stated that the body of literature on the effectiveness of decision aids for older adults was still in its early stages and suggested *that future research should expand on the design, application and evaluation of decision-making aids for older and more vulnerable adults*. However, the role of self-efficacy in decision making maybe mediated by preparation rather than knowledge [12].

Health literacy refers to an individual having the skills and knowledge about health and health care; an ability to find, understand, interpret and communicate health information, to make decisions about their health and to know when to seek appropriate care [13]. Low levels of health literacy are associated with poorer treatment outcomes, including low compliance with medication, increased admissions to emergency departments, lower ability to interpret labels and health messages, reduced health status, and increased mortality among the elderly [14]. With increasing numbers of older people accessing health information on the Internet [15], it is important that individual have the skills to assess the quality of information they are accessing.

It is important that health consumers are able to understand the current evidence, or lack thereof, surrounding CM and the potential risks and benefits. Positive outcomes of CM, therefore are dependent on good health literacy skills [16]. This could be of particular concern due to a higher prevalence of polypharmacy arising from the treatment of complex chronic health conditions [17]. This concern may increase the risk of potential CM-drug interactions, and is further complicated by limited disclosure of CM use between consumers and their health care providers. Non-disclosure rates among those using CM to their health care providers has been reported to be as high as 70% [18]. Disclosure and communication about CM is essential for achieving optimal treatment outcomes.

A study with Australian seniors found differing competencies relating to CM health literacy [19]. Based on these study findings, we developed an intervention to improve skills and ability in identifying good and reliable sources of CM information, resolving conflicting information, and accessing and using a diverse range of CM information sources to find information that is current and can be used to guide seniors’ CM decision with their health care providers. The aim of this study was to determine the effectiveness of a CM educational intervention to increase older adults’ decision self-efficacy, decision making preparedness and health literacy. The primary hypothesis was that participants receiving a web/DVD education intervention compared with a booklet only group would demonstrate an increase in decision self-efficacy. The secondary hypothesis was that participants receiving a web/DVD education intervention compared with a booklet only group would demonstrate an increase in health literacy. Outcomes were assessed at 3 weeks, and at 2 months follow up from trial entry.

## Methods

### Participants and setting

We recruited participants from a community setting comprised of retirement villages and community groups, including senior citizen clubs and associations, based in Sydney, Australia. In Australia, a retirement village is made up of housing for people aged over 55 years who are able to live independently, with many villages offering some health care services, leisure facilities and social clubs. Inclusion criteria included; aged 65 years and older, with access to the Internet or a DVD player or a computer, and providing informed written consent. Exclusion criteria included; unable to communicate in English, or living in a long-term care facility.

### Study design

We conducted a parallel randomised controlled trial delivered online (using a purpose-built web-site) or by a DVD plus booklet versus a control group (written booklet only) [20] delivered over 3 weeks, with a 2 month follow up. The study was conducted between July 2016 and January 2017.

### Sample recruitment and retention

Participants were recruited through letter box drops, and promotional talks by the investigators and the trial co-ordinator. At these visits, expressions of interests were gathered and participant information and consent forms were made available. A mutually convenient time was then made to obtain informed consent and to complete baseline questionnaires. No incentives were provided to potential study participants. Following randomisation, the trial co-ordinator met with each participant to ensure they were able to access the website using the password, and to navigate the modules, or ensure navigation with the DVD was satisfactory. To minimise attrition, all groups received a telephone call mid-way through the intervention to ensure they were continuing to access the resources, and to address any difficulties participants were experiencing.

### Interventions

Delivery of multi-media education programs have been shown to offer advantages over the traditional information delivery methods including spoken communication and supplementary written information with regards to improving knowledge and skills [21], and can overcoming difficulties with low literacy skills [22]. Studies have also shown that learning is improved when material is presented as an audio-visual rather than visual alone format [23, 24]. The delivery of the intervention was, therefore, comprised of a multi-media (web-based/DVD) intervention, and booklet. The web based/DVD format comprised of audio-visual delivery and an interactive format, and the booklet comprised of visual information alone. We therefore hypothesised the web based/DVD format would be more effective with improving decision self-efficacy skills, and health literacy compared with a booklet alone.

The intervention was informed by an understanding of nine health literacy concepts utilised in the described in the Ophelia Project [25]. These concepts include “sufficient information to manage health, social support for health, skills to appraise health information, ability to engage with health care providers, capacity to navigate the health care system, ability to find good health information, and sufficient understanding of health information to know what to do with it”. The intellectual content of the intervention was adapted from the Complementary Medicine Education and Outcomes (CAMEO) research program (cameoprogram.org) [26]. CAMEO is designed to support patients and their families to make safe and informed decisions about CM. The theoretical foundation of CAMEO is based on two decision-making theories; the model of Shared Decision Making (SDM) [27], the Ottawa Decision Support Framework (ODSF) [28]. The SDM model involves patients in decision making to develop patient centred, preference decisions that improve knowledge, and lower decisional conflict. The intent of the ODSF framework is to improve the quality of decision making by clarification of values, and providing additional information offered through values-based choices. The theoretical framework of the Supportive Care Framework [29] provided a framework to tailor information and decision support strategies addressing basic and complex CM needs. CAMEO was modified for use in the Australian context, and adapted for use with an older population. The SDM theory and ODSF framework are aligned with our selected outcome measures, and have been previously used in an evaluation of CAMEO [26]. The educational intervention has been informed by our preliminary research [19], highlighting areas of lower health literacy.

### Group 1: DVD/web format

This resource comprised of five modules covering;

- Module 1-*Complementary Medicine-The Evidence.* This module provides scientific evidence related to the health benefits of CM, its indications, and details of various evidence-based CM that are widely practiced globally.
- Module 2-*Finding and Evaluating Complementary Medicine Evidence*. This module provides an introduction to scientific evidence and how to find research-based studies about CM. It describes databases that are available to find research, how to conduct a search, and how to use the available evidence in making an informed choice about CM.
- Module 3-*Decision Making – Complementary Medicine.* In this module, advice is provided regarding how to bring together the information they have obtained from earlier modules, aligning this with their goals and values, and how to have discussions with relevant key people to make an informed decision about the use of CM. The universally employed SCOPED framework [30], (i.e., SCOPED stands for: Situation, Choices, Objectives, People, Evaluation, and Decisions) has been incorporated to assist the participant in their decision making.
- Module 4-*Working with Complementary Medicine Practitioners*. This module explains the role of conventional health care providers and CM practitioners, and the importance of, and how to, disclose CM use with conventional health providers. Guidance is provided regarding the regulatory framework for CM practitioners in Australia, how to find a professionally accredited CM practitioner, and practical tips and questions to ask to guide the selection of a CM practitioner.
- Module 5-*Monitoring Complementary Medicine Decisions*. This module explains the need to monitor one’s health following the use of CM therapies. It provides guidance on certain criteria that should be utilised in respect to monitor one’s health and the safety of CM and the procedure (including contact details) of adverse events reporting for CM therapy and services in Australia. There is a final section that includes two case studies of individuals exploring self-care and use of CM that draws on the detailed information presented in the modules.

Participants were invited to watch the five-module intervention in their home by either accessing the study website, or viewing the DVD on a DVD player or computer, over a three-week time period. Each module took approximately 30 min to complete, and completion of two modules per week were recommended. Participants also received a copy of the booklets distributed to the control group.

### Group 2: control group

The active control group was comprised of two booklets that summarised content from modules one, three, and five, and the case studies. The content focuses on presenting information on; evidence-based CM modalities, guidance to sourcing reliable CM information, how to make decisions about evidence-based CM, why it is important to monitor and evaluate the use of CM, and details about how to discuss CM use with your health care provider. A second booklet provided written examples of the two case studies, and applying the information in practice. The booklet text was written in 18 point Arial font and at a 6th grade reading level. Paced reading was encouraged over the three-week intervention.

### Randomisation and blinding

The randomisation sequence was computer generated by the Sealed Envelope online randomisation service (http://sealedenvelope.com) with the codes concealed in sealed, opaque envelopes. Participants were randomised in a 1:1 ratio, in blocks of eight to either Group 1: web/DVD resource plus booklet, or Group 2: control booklet only. Participants were not blind to their group allocation; however, the study analyst was blind to study group during analysis, and the codes were broken following statistical analysis. AK recruited and allocated participants to their group.

### Outcomes

Outcomes were assessed at baseline, at 3 weeks and at 2 months from baseline. The Decision Self-Efficacy scale [31] was used to assess the primary outcome, namely decision-making skill and was developed to measure self-confidence or belief in one’s ability to make decisions. This valid and reliable scale [28] is comprised of 11 items and assessed confidence along a five-point scale ranging from “not at all confident” to “very confident”. The psychometric properties report an alpha coefficient of 0.92, and the scale has been shown to be correlated with select subscales of the Decisional Conflict scale (DSC) (i.e., feeling informed (0.47) and supported (0.45) sub-scales) [28]. Scores on the scale are converted to a 0 to 100 scale. Scores range from 0 (extremely low self-efficacy) to 100 (extremely high self-efficacy).

### Secondary outcomes

The health literacy of participants was evaluated using the Health Literacy Questionnaire (HLQ) [25]. The HLQ is grouped into nine domains including: (1) feeling understood and supported by health care providers; (2) having sufficient information to manage personal health; (3) an ability to actively manage personal health; (4) social support for health; (5) appraisal of health information; (6) ability to actively engage with health care providers; (7) navigating the health care system; (8) ability to find good health information; and (9) understanding health information well enough to know what to do. Participant’s indicate their response along a four-point scale with response options ranging from “very difficult” to “very easy”, or along a five-point scale was ranging from “strongly agreed” to “strongly disagreed.” The HLQ has strong psychometric properties [25]. Health literacy scores will be calculated using a scoring algorithm for the HLQ version 1 (dated 2012). The algorithm produces unweighted scale scores for the nine scales of the HLQ, with the final score for each subscale being an average score across all items forming the scale. For missing values, this program uses an algorithm to impute missing values. For scales with four to five items, two missing values can be imputed. For scales with six items, three missing value can be imputed, and if more responses among the scale items are missing, scale score cannot be computed.

The Preparation for Decision-Making scale assesses participant’s perception of how useful a decision aid or other decision support intervention is in preparing the individual to communicate with their health care provider at a consultation focused on making a health decision [32]. This scale can only be administered post intervention. The scale consists of 10 statements rated along a five-point scale from “not at all” to “a great deal”, and has undergone reliability and validity testing [32]. This scale has shown significant correlation with the feeling informed (*r* = − 0.21, *p* < 0.01) and supported (*r* = − 0.13, *p* = 0.01) DSC subscales [28]; and discriminates significantly between participants who did and did not find the decision aid helpful (*p* < 0.0001). Alpha coefficients for internal consistency ranged from 0.92 to 0.96. The scale is strongly unidimensional and Item Response Theory analyses demonstrated that all 10 scale items perform well [32]. The items are summed, scored and converted to a 0–100 scale. Higher scores indicate a higher perceived level of preparation for decision making.

Other data collected at baseline data included; socio-demographic (age, gender, place of birth, education status, employment status, ethnicity, English skills, Medicare and private health insurance), and health characteristics, health behaviour, and lifestyle including CM use, sources of information, Internet skills, health literacy status, and decision making. We also sought participants’ views on the educational resources at the end of the intervention, and use of the resources at the follow up at 2 months.

### Sample size

The sample size was calculated using GPower [33], and was based on a ‘sample size effect’ drawn from published data [34]. It was estimated that a moderate effect size would be obtained (i.e. Cohen D = 0.5) for the primary endpoint only, with improved decision self-efficacy between groups at the end of the three-week intervention. With alpha value set at 0.05 and power at 0.8 (i.e., 80% chance that the expected effect size would be significant), a minimum sample size of 64 per group, with rounding to 70 per group was estimated. Allowing for 20% attrition, a total sample size of 168 participants was required for this trial (i.e., 84 per group).

### Statistical analysis

Baseline characteristics were summarised using counts and percentages for categorical variables and means and standard deviations for numeric variables. Pearson’s chi-square (*X*^2^) and analysis of variance (ANOVA) were used to identify the differences between intervention groups for the categorical and continuous variables, respectively, at the end of the intervention. Secondary analyses examined changes within group over time using a repeated measures ANOVA from baseline to the end of the two-month follow up adjusting for Internet usage at baseline, and using Sidak to correct for multiple comparisons [35]. There was missing data from 22 subjects who either died or withdrew prior to the end of the intervention, and they were excluded from the analysis. All analyses were conducted using SPSS statistical software, version 22. Differences in outcomes were expressed as mean differences (MD), with 95% confidence intervals (CI). We also reported partial Eta-squared to examine effect sizes. The significance level was set to less than 0.05.

## Results

**Fig. 1:**
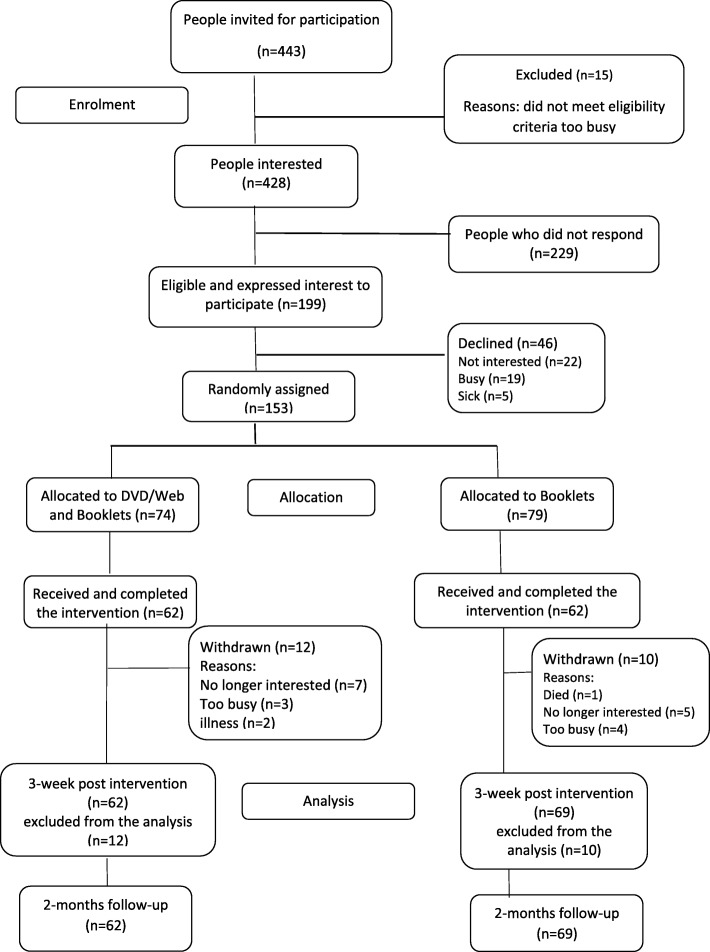
Consort flow chart for trial participants

Of the 443 individuals invited to participate, 199 met the eligibility criteria of which 153 were randomised. Follow up at 3 weeks and 2 months was completed by 131 participants. There was a 14% (*n* = 22) attrition rate (Fig. 1). Of those randomised, 66% were females and the mean age was 76.0 years (Table 1). Most participants reported having very good or good health, and the most common health problem was arthritis (Table 2). At baseline, most characteristics were similar between the groups, with the exception that participants in the web/DVD group used the Internet and media as a source of information more often than the control. As such, primary and secondary study outcomes were adjusted for previous Internet usage. Our population was compared with data from the 2011 Australian Census. We found our sample had a greater representation of women, participants born in Australia, retired and an under-representation of Aboriginal and Torres Strait Islander (ATSI) peoples.

**Table 1:** Socio-demographics of trial participants

Demographics (*n* = 153)	Web/DVD *n* = 74 (%)	Booklet *n* = 79 (%)	Data from ABS^b^ n (%)	*P* value
Sex				
Male	27 (36.5)	24 (30.4)	1,378,446 (45.76)	0.00
Female	47 (63.5)	55 (69.6)	1,633,846 (54.24)	
Age (M SD)^a^	76.4 (6.9)	76.2 (7.7)		
Marital *status*				
Single	4 (5.4)	10 (12.8)	139,550 (4.63)	0.06
Married/defacto	43 (58.1)	42 (43.8)	1,731,174 (57.47)	
Widowed	16 (21.6)	18 (23.1)	774,645 (25.72)	
Divorced/Separated	11 (14.9)	8910.3)	366,912 (12.18)	
Live alone	30 (40.5)	32 (41.0)		
Country of birth				
Australia	52 (70.3)	51 (65.4)	1,787,070 (59.33)	0.03
United Kingdom	13 (17.6)	12 (15.4)		
Other	9 (12.2)	15 (19.2)		
Missing		1 (1.2)		
Speak English at home	74 (100.0)	79 (100.0)		
Aboriginal or Torres Strait Islander	2 (2.7)	1 (1.3)	184,105.00 (6.11)	0.03
Highest level of education	7 (9.5)	15 (19.0)		
High school (not completed)	19 (25.7)	19 (24.1)		
High school (completed)	21 (28.4)	15 (19.0)		
TAFE/Trade	19 (25.7)	21 (26.6)		
University undergraduate	8 (10.8)	9 (11.4)		
Employment status				
Retired	69 (93.2)	70 (88.6)	2,433,731 (80.79)	0.00
Annual household income/year				
< $20,000	7 (9.6)	19 (24.1)		
$20,000-39,999	31 (42.5)	29 (36.7)		
$40,000-59,999	11 (15.1)	8 (10.1)		
$60,000-75,999	5 (6.8)	7 (8.9)		
> $80,000-99,999	3 (3.1)	1 (1.3)		
Prefer not to answer	16 (21.9)	15 (19.0)		
Use of the internet	70 (94.6)	61 (78.2)		
Own no device	5 (6.9)	15 (19.2)		
Own a tablet	42 (56.8)	33 (42.3)		
Laptop	35 (47.3)	36 (46.2)		
Desktop	36 (48.6)	30 (38.5)		
Smart phone	32 (43.2)	36 (46.2)		

^a^ Mean and standard deviation

^b^ Australian Bureau of Statistics

**Table 2:** Health characteristics at baseline of study participants

Demographics (*n* = 153)	Web/DVD *n* = 74 (%)	Booklet *n* = 79 (%)
Private health insurance	53 (72.6)	62 (78.5)
Have a health care card	50 (69.4)	61 (79.2)
Current health status		
- No health problem	15 (20.3)	11 (13.9)
- Arthritis	35 (47.3)	41 (51.9)
- Heart problems	19 (25.7)	21 (26.6)
- Back pain	16 (21.6)	25 (31.6)
- Asthma	11 (14.9)	11 (13.9)
- Diabetes	10 (13.5)	9 (11.4)
- Cancer	8 (10.8)	10 (6.5)
- Stroke	1 (1.4)	2 (1.3)
Attended a hospital emergency department in past 12 months	14 (18.9)	16 (20.3)
Current health status		
- Excellent	2 (2.7)	5 (6.3)
- Very good	29 (39.2)	29 (36.7)
- Good	27 (36.5)	36 (45.6)
- Fair	13 (17.6	9 (11.4)
- Poor	3 (4.1)	0 (0.0)
- Non-smoker	71 (95.9)	77 (97.5)
Alcohol consumption in past 12 months	52 (70.3)	58 (73.4)
Frequency of alcohol		
- 1–2 days/week	7 (13.4)	17 (29.3)
- 3–4 days/week	5 (9.6)	8 (13.7)
- 5–6 days/week	5 (9.6)	3 (5.1)
- Everyday	8 (15.3)	5 (8.6)
- 1–3 days/month	8 (15.3)	11 (18.9)
- Few times/year	19 (36.5)	14 (24.1)
Amount		
- 1–2 drinks	43/49 (87.8)	44/56 (78.6)
- 3–10+ drinks	6/49 (12.2)	12/56 (21.4)
Regular physical activity > 1/week	64 (86.5)	58 (75.3)

### Primary outcome

Adjusted and unadjusted analyses are reported. Decision self-efficacy did not differ between groups at the end of the intervention (MD 3.8, 95% CI -2.0 to 9.6, *p* = 0.20), or at the two-months follow up (MD 2.7, 95% CI-2.8 to 8.3, *p* = 0.33) (Table 3). Decision self-efficacy increased over time between baseline and two-months follow up (MD 11.9, 95% CI 8.1 to 15.6, *p* < 0.001, partial Eta^2^ = 0.306).

**Table 3:** Outcome: decision making by study groups at the end of the intervention and at follow up

Overall score/100	web/DVD mean SD	Booklet mean SD	Unadjusted treatment effect (95% CI)	Unadjusted P	Adjusted treatment effect (95% CI)	Adjusted P and (partial Eta^2^)
Primary *Decision elf Efficacy Scale* End of the intervention (*n* = 62/69)	85.4 (13.0)	81.6 (20.9)	3.8 (−2.1 to 9.7)	0.21	3.8 (−2.0 to 9.6)	0.20 (0.01)
2 months follow up (*n* = 62/69)	87.2 (11.9)	82.8 (19.9)	4.4 (−1.15 to 9.95)	0.12	2.7 (−2.8 to 8.3)	0.33 (0.01)
Secondary *Preparation for Decision Making Scale* End of the intervention (*n* = 62/69)	64.9 (25.8)	58.3 (27.7)	8.6 (−2.5 to 15.7)	0.16	6.3 (−2.8 to 15.4)	0.18 (0.001)
2 months follow up (*n* = 62/69)	60. (28.1)	58.0 (26.2)	2.1 (−7.2 to 11.4)	0.66	0.2 (−9.1 to 9.5)	0.97 (0.013)

*CI* confidence interval, treatment effect is mean difference

### Secondary outcomes

There were no differences in the Preparation for Decision-Making scale between groups at the end of the intervention (MD 6.3, 95% CI-2.8 to 15.4) and at the two-months follow up (MD 0.2, 95% CI-9.1 to 9.5) (Table 3). The health literacy domains between groups did not differ between groups at the end of the intervention and at the two-months follow up (Table 4).

**Table 4:** Health Literacy Questionnaire domain scores between groups at the end of the intervention and at follow up

Post intervention *n* = 62/69 Follow up *n* = 62/69	Web/ DVD mean SD	Booklet mean SD	Unadjusted treatment effect (95% CI)	Unadjusted P	Adjusted treatment effect (95% CI)	Adjusted P	Partial Eta^2^
^a^Having sufficient information to manage my health	3.1 (0.4)	3.0 (0.5)	0.1 (−0.0 to 0.2)	0.20	0.0 (− 0.1 to 0.2)	0.43	0.009
	3.1 (0.5)	3.0 (0.4)	0.1 (− 0.0 to 0.2)	0.25	0.1 (− 0.0 to 0.2)	0.25	
Feeling understood and supported by health care providers	3.3 (0.6)	3.3 (0.5)	0.0 (−0.1 to 0.1)	1.00	0.0 (−0.1 to 0.2)	0.84	0.007
	3.4 (0.5)	3.3 (0.5)	0.0 (−0.1 to 0.2)	0.44	0.0 (− 0.1 to 0.2)	0.74	
Actively managing my health	3.1 (0.4)	3.0 (0.5)	0.1 (−0.0 to 0.2)	0.20	0.0 (−0.1 to 0.2)	0.38	0.006
	3.1 (0.5)	3.1 (0.4)	0.0 (−0.1 to 0.2)	0.56	−0.0 (− 0.2 to 0.1)	0.65	
Social support for health	3.1 (0.4)	3.0 (0.5)	0.1 (−0.0 to 0.2)	0.20	0.0 (−0.0 to 0.31)	0.21	0.01
	3.2 (0.5)	3.1 (0.5)	0.1 (−0.0 to 0.2)	0.27	0.1 (−0.0 to 0.2)	0.23	
Appraisal of health information	2.9 (0.4)	3.0 (0.6)	−0.1 (− 0.2 to 0.0)	0.26	− 0.0 (− 0.2 to 0.1)	0.40	0.000
	3.0 (0.4)	3.0 (0.5)	0.0 (− 0.1 to 0.1)	1.00	−0.0 (− 0.2 to 0.1)	0.74	
^b^Ability to actively engage with health care providers	4.0 (0.6)	4.0 (0.6)	0.0 (−0.2 to 0.2)	1.0	−0.0 (− 0.2 to 0.1)	0.79	0.005
	4.1 (0.6)	4.1 (0.5)	0.0 (−0.1 to 0.2)	0.70	−0.0 (0.2 to 0.1)	0.92	
Understand health information well enough to know what to do	4.2 (0.5)	4.1 (0.5)	0.1 (−0.0 to 0.2)	0.25	−0.0 (− 0.2 to 0.5)	0.75	0.007
	4.3 (0.5)	4.2 (0.5)	−0.0 (− 0.0 to 0.2	0.17	0.0 (− 0.1 to 0.2)	0.50	
Ability to find good information	3.9 (0.5)	3.8 (0.6)	−0.1 (− 0.0 to 0.2)	0.30	− 0.0 (− 0.2 to 0.1)	0.92	0.007
	4.0 (0.4)	3.8 (0.6)	0.1 (− 0.0 to 0.2)	0.20	0.0 (− 0.1 to 0.2)	0.52	
Navigating the health care system	3.9 (0.5)	3.9 (0.6)	0.0 (−0.1 to 0.1)	1.00	−0.0 (− 0.2 to 0.1)	0.55	0.000
	3.9 (0.4)	3.9 (0.5)	0.0 (− 0.1 to 0.1)	0.81	−0.0 (− 0.2 to 0.1)	0.72	

^a^How strongly do you agree or disagree (1–4 point scale), ^b^ How easy or difficult are the following tasks for you to now cannot do to very easy (1–5 point scale)

CI, confidence interval, treatment effect is mean difference

### Participant’s view of the resources and use following the intervention

Participants held very positive views regarding the content of the education resources (Table 5). At the two-months follow up, 28 (45.2%) web/DVD group participants, and 21 (30.0%) booklet only group participants reported independently using the resources (*p* = 0.08). Use of the website content described in the resources was high in both groups, reported by 85% in the web/DVD group, and 71% in the booklet only group.

**Table 5:** Views on the resource content by study group at the end of the intervention

Web/DVD *n* = 64	Poor/Fair		Good/Excellent		*P* value
	Web/DVD	Booklet	Web/DVD	Booklet	
Booklet only *n* = 69	n %	n %	n %	n %	
Understanding evidence	9 (14.5)	12 (17.4)	53 (85.5)	57 (82.6)	0.65
Where to find reliable information	9 (14.5)	10 (14.5)	53 (85.5)	59 (85.5)	0.99
Questions to ask about research articles	13 (21.0)	15 (21.7)	49 (79.0)	54 (78.3)	0.91
Reliable internet resources	11 (18.6)	18 (26.9)	48 (81.4)	49 (73.1)	0.27
Questions to ask about websites	9 (15.0)	18 (27.3)	51 (85.0)	48 (72.7)	0.09
Finding credible resources	10 (16.1)	21 (30.9)	52 (83.9)	47 (69.1)	0.04
Making an informed decision	12 (19.4)	12 (17.4)	50 (80.6)	57 (82.6)	0.77
Tips to working with a CM practitioner	14 (23.7)	14 (20.6)	45 (76.3)	54 (79.4)	0.67
Evaluating your use of CM and self help	16 (27.6)	15 (22.1)	42 (72.4)	53 (77.9)	0.47

### Secondary analyses

An adjusted analysis examining group changes from baseline to two-months follow up found no significant difference for any outcome between the groups over time. A significant improvement in decision self-efficacy was found within each group (Table 6), (web/DVD mean 11.7, 95% CI 8.1 to 15.3, *p* = 0.001 partial Eta^2^ = 0.36; booklet mean 12.1, 95%CI 7.1 to 17.3, *p* = 0.001, partial Eta^2^ = 0.26). There were no further changes for the web/DVD group over time. For the booklet group, there were improvements on four health literacy domains; feeling understood by health care providers, appraisal of information, ability to engage with healthcare providers, and understanding health information well enough to know what to do with it.

**Table 6:** Change in study outcomes over time within group adjusted for internet usage

	DVD/Web intervention *n* = 62				Booklet only *n* = 69			
	Mean (SD)	95% CI	*P* value	Partial Eta^2^	Mean (SD)	95% CI	*P* value	Partial Eta^2^
Decision self-efficacy	11.7 (17.3)	8.1 to 15.3	0.001	0.362	12.1 (13.5)	7.0 to 17.1	0.001	0.26
Health Literacy Questionnaire: domains								
Having sufficient information to manage my health	0.1 (0.5)	−0.0 to 0.2	0.14	0.043	0.1 (0.5)	−0.0 to 0.2	0.09	0.01
Feeling understood and supported by health care providers	0.0 (0.4)	−0.0 to 0.2	0.46	0.022	0.1 (0.5)	0.0 to 0.3	0.01	0.037
Actively managing my health	0.0 (0.5)	−0.1 to 0.1	0.82	0.003	0.1 (0.5)	−0.0 to 0.24	0.14	0.045
Social support for health	0.0 (0.5)	−0.0 to 0.1	0.40	0.029	0.1 (0.5)	−0.0 to 0.2	0.13	0.023
Appraisal of health information	0.0 (0.5)	−0.0 to 0.1	0.50	0.032	0.1 (0.4)	0.0 to 0.3	0.03	0.033
Ability to actively engage with health care providers	0.0 (0.5)	−0.1 to 0.1	0.71	0.016	0.1 (0.4)	0.0 to 0.3	0.03	0.039
Understand health information well enough to know what to do	0.0 (0.5)	−0.0 to 0.2	0.18	0.053	0.1 (0.4)	0.0 to 0.3	0.02	0.084
Ability to find good information	0.0 (0.5)	−0.0 to 0.2	0.27	0.014	0.1 (0.5)	−0.0 to 0.2	0.05	0.032
Navigating the health care system	0.0 (0.5)	−0.1 to 0.1	0.71	0.009	0.1 (0.5)	−0.0 to 0.3)	0.06	0.022

## Discussion

This is the first study to examine the effectiveness of a CM education intervention to improve older adults’ decision making and health literacy. We found no significant differences on any outcome between the delivery of information using a website/DVD and booklet versus booklet only, suggesting no particular format was more effective than the other with improving outcomes. Both groups of participants found the resources useful, and their scores suggest the resources better prepared them to communicate with their practitioner at a consultation focused on making a health decision. Over time, decision self-efficacy improved for both groups, and for the booklet only group, health literacy scores improved from baseline to the follow up on four health literacy domains. The web/DVD group reported greater Internet use at baseline and when adjusting for this variable, no improvements were seen in health literacy over the course of the study.

The improvements in decision self-efficacy within both groups from baseline to 2 months are interpreted as large effects (> 0.14) from the partial Eta squared statistics [36], and are meaningful important differences. Large effects were also found for seven of the nine health literacy domains, and medium effects (> 0.13) for two health literacy domains. The effect sizes were small (0.01) for the booklet only groups over this time period; however, these results may have arisen from the repeated measure, and this finding should be interpreted with some caution.

Studies to improve health literacy of older people have been conducted with people living with chronic health conditions. Shreffler-Grant et al. state that without adequate CM health literacy, older consumers may not understand health care choices that may benefit or harm them [37]. A recent Australian study of patients with diabetes, tested a tailored, self-management education intervention to improve health literacy [38]. This study of 113 clients with a mean age of 75 years, reported mean HLQ scores similar to our study population. Following the delivery of the diabetes education intervention pre and post HLQ, data demonstrated small but non-significant increases on two HLQ domains, a finding similar to our study. Studies examining decision-making aids among older people are also limited. An evaluation of online and offline sources of health information examined decision self-efficacy in a random sample of 250 older adults aged 50–92 years in the United States [39]. Low self-efficacy scores were found for this population (mean 29.93; SD 3.41), with no difference between groups. Study findings may have been influenced by the method of sampling used, resulting in the selection of a socio-demographic group that has low baseline decision self-efficacy. The Decision-Making Preparedness scale has been used in one Australian study evaluating an interactive decision aid comprising of a video booklet and a web-based tool. This study of 360 participants with osteoarthritis [40], was comprised of 41% of participants aged over 65 years. Scores on this scale were high (70 and 74 in the two groups), which may have reflected the fact that over half of participants were aged less than 65 years and attained a higher educational achievement. Our study findings are comparable to a study of carers of persons with dementia who participated in a web-based e-health support service, in which the mean score on the Preparation for Decision Making scale was 67.9 [41]. In summary, our findings in response to decision making are similar or better to comparable populations from other studies. Overall, we found older Australians engaged with the choice of decision support resources presented, with no clear preference stated.

There are several study limitations. Comparison of our population with national 2011 Census data suggests our results may not be generalisable to a wider population of senior Australians. Our control was an active control comprised of a booklet and all participants received some information; the effectiveness of the resources could be determined from the inclusion of a no intervention arm in future studies. There is also the potential for some contamination between groups, particularly for those participants residing in retirement villages. We do not know if resources were shared between participants allocated to different groups. The study sample size was estimated to detect a moderate difference in outcomes between the two groups, however, our findings suggest the effect size was smaller and we were underpowered to detect any differences between groups. We did not evaluate participants’ learning style, and an understanding of this may or may not have influenced our findings. Nevertheless, our results provide evidence that use of multi-media education programs offer advantages over traditional information delivery [21].

Health literacy is important as health information and health systems increase in complexity. The greater usage of the Internet at baseline in the DVD/web group suggests that seniors may have increasing computer literacy and familiarity with seeking information. The Australian Commission on Safety and Quality in Health Care Report [42] recommends research that addresses health literacy in the Australian context and for the need for an evaluation of programmes. This study contributes to this gap. However, our results also highlight further work remains to improve health literacy and senior Australians’ capacity to make decisions about their health and health care. Future research should seek greater representation from seniors with low socio-economic status, culturally and linguistically diverse populations and geographically remote communities. The inclusion of a wait list or no intervention arm would allow exploring random effects which may or may not explain benefits from the interventions. There is also a need to undertake research to explore seniors’ experiences of navigating the health care system in general, including conventional health and allied health services as well as CM, and their ability to access evidence-based information.

## Conclusion

The effectiveness of CM resources on decision self-efficacy and health literacy did not differ by the mode of delivery of the resources by DVD, website or booklet. Participants found the resources useful, and rated the content as good or excellent. Delivery of CM education using booklets, DVD and a website information for older Australians has the potential for wider application.

## Abbreviations

ANOVA: Analysis of variance
ATSI: Aboriginal and Torres Strait Islander
CAMEO: Complementary Medicine Education and Outcomes
CI: Confidence interval
CM: Complementary medicine
DSC: Decisional Conflict scale
HLQ: Health literacy questionnaire
MD: Mean difference
ODSF: Ottawa Decision Support Framework
SCOPED: Situation, Choices, Objectives, People, Evaluation, and Decisions
SD: Standard deviation
SDM: Shared Decision Making
